# Supplementary material for: Acceptance and compliance with micronutrient powder (MNP) among children aged 6–23 months in northern Nigeria
Source: PLOS Glob Public Health. 2022 Oct 17;2(10):e0000961. doi: 10.1371/journal.pgph.0000961 (PMC10022258; doi:10.1371/journal.pgph.0000961)
Supplement: S1 Dataset — (ZIP) [file pgph.0000961.s008.zip › S8_Dataset/Direct Observations.zip!/Direct Observations/020705_A_SO_AD_2_DO.pdf]

1.1.1 Instrument F –Direct Observation Form

<<Instrument F Direct Observation Form>>

PI Name: PragyaMathema,Stephen Kodish
Study Title: Formative research to develop an infant and young child feeding (IYCF) intervention with micronutrient powder (MNP) for children aged 6 – 23 months
PI Version/Date: Version1.0/January 24, 2015

Date: _July 02, 2015_____________Location: _Bolki community, LGA -Song

Data Collector: Aisha Aliyu Dahiru________ Participant ID: __ Mud House with Zinc door

Main goals of this direct observation are to answer the following questions:

1. In this household, what would be the best way(s) to give nutritional supplements to young children? Why?

2. In this household, describe the food sharing that you observe throughout the meal. Are there any predictable patterns?

3. In this household, who is favored and how is this favoritism expressed during meal preparation and eating?

(TEAR OFF AT PERFORATED DOTTED LINE BELOW)

PARTICIPANT ID: __

Household Composition Table (Fill out as you do data collection)

INITIALS	SEX (M/F)	AGE (estimated)	Relationship to household	Description
W	F	28 YRS	Mother	Wearing yellow shirt with brown wrapper.
C	F	17 MONTHS	Daughter (baby girl)	Wearing brown dress with a flower on it.
C1	M	6 Yrs	Older son	Wearing checked shirt with brown trouser
C2	F	4 Yrs	Older daughter	Wearing pink dress

PARTICIPANT ID: __________

TIME	ACTOR INITIALS	RECIPIENT INITIALS	WHAT HAPPENED	FOOD	Reactivity
8.00	W		Washing the utensils		
8:05	W		Washing utensils		
8:10	W		Washing utensils		
8:15	W	C	Carrying baby on her back		
8:20	W	C	Bringing out large utensil with hoe from the room		
8:25	W	C	Walking to the farm		
8:30	W		Walking to the farm		
8:35	W		Walking to the farm while carrying child on her back		
8:40	W	C	Carrying child on her back walking to the farm		
8:45	W	C	Carrying child on her back walking to the farm		
8:50	W	C	Carrying baby on her back walking to the farm		
8:55	W	C	Carrying baby on her back walking to the farm		
9:00	C		Child is sleeping on the mother’s back		
9:05	W	C	Carrying on the back walking to the farm		

TIME	ACTOR INITIALS	RECIPIENT INITIALS	WHAT HAPPENED	FOOD	Reactivity
9:10	C		Sleeping on mother's back		
9:15	W	C	Lying baby down on a wrapper under the tree in the farm		
9:20	W		Placing the large utensil near the child		
9:25	C		Sleeping under the tree in the farm		
9:30	W		Picking the hoe from the utensil near the baby		
9:35	W		Farming		
9:35	C		Sleeping under the tree in the farm		
9:40	C		Sleeping under the tree in the farm		
9:45	W		Farming		
9:50	W		Checking on the child. the child is still sleeping		
9:55	W		Farming		
10:00	W		Farming		
10:05	C		Sleeping		
10:10	C		Sleeping under the tree in the farm		
10:15	W		Farming		
10:20	W	C	Checking on the child. The child is still sleeping		
10:25	W		Resting under the tree near the child sitting		
10:30	W		Lying down beside the child under the tree		
10:35	C		Still sleeping under the tree		
10:40	W		Sleeping near the child		
10:45	W		Sleeping near the child under the tree		
10:45	C		Sleeping under the tree		
10:50	W		Sitting under the tree beside the child		
10:55	W		Drinking water from a cup	Water	
10:55	W	C	Carrying the child on her lap		
11:00	W	C	Breastfeeding the child sitting on her lap	Breast milk	
11:05	W	C	Breastfeeding the child	Breast	

TIME	ACTOR INITIALS	RECIPIENT INITIALS	WHAT HAPPENED	FOOD	Reactivity
				milk	
11:10	W	C	Breastfeeding the child	Breast milk	
11:10	W	C	Breastfeeding the child	Breast milk	
11:15	W	C	Breastfeeding the child	Breast milk	
11:20	C		Playing under the tree		
11:20	W		Farming		
11:25	C		Playing under the tree with the large utensil the mother placed beside him		
11:25	W		Farming		
11:30	C		Sitting inside the large utensil, playing with cups and spoons.		
11:35	W		Farming		
11:40	W		Sitting under the tree near child		
11:40	C		Still playing inside the utensil		
11:45	W		Washing hands with water		
11:45	W		Serving kunu from flask into a cup.	Kunun gyada	
11:45	W		Stirring the kunu to cool	Kunun gyada	
11:50	W	C	Feeding the child kunu with a spoon. The child is anxiously taking the kunu	Kunun gyada	
11:51	W	C	Feeding the child kunu with a spoon. The child is willingly drinking with enthusiasm	Kunun gyada	
11:52	W	C	Feeding the child kunu with a spoon the child is drinking with enthusiasm	Kunu gyada	
11:53	W	C	Still feeding the child kunu	Kunu gyada	
11:54	W	C	Still feeding the child kunu	Kunun gyada	
11:55	W	C	Washing the child hands and mouth with water		
11:55	W	C	Carrying child on her lap		
12:00	W	C	Giving child water to drink from a small cup	Water	

TIME	ACTOR INITIALS	RECIPIENT INITIALS	WHAT HAPPENED	FOOD	Reactivity
12:00	W		Drinking water from the same cup she gave the child to drink	Water	
12:05	C		Child playing on the ground under the tree		
12:05	W		Farming		
12:10	C		Playing on the ground under the tree		
12:15	W		Farming		
12:15	C		Playing on the ground under the tree		
12:20	C		Following the mother as she farms		
12:25	C		Playing near the mother as she farms		
12:30	W		Taking a break to drink water from the plastic container	Water	
12:30	W	C	Giving the child water to drink from a cup. The cup the child played with but was rinsed by the mother	Water	
12:35	W		Continue farming		
12:35	C		Playing with the large utensil under the tree		
12:40	C		Lying down on the wrapper spread under the tree		
12:45	C		Sleeping under the tree		
12:50	W	C	Checking on the child		
12:55	W		Continue farming		
1:00	W		Farming		
1:05	C		Sleeping under the tree		
1:10	C		Still Sleeping		
1:15	W		Taking a break from farming		

TIME	ACTOR INITIALS	RECIPIENT INITIALS	WHAT HAPPENED	FOOD	Reactivity
1:15	W		Washing hands with water		
1:20	W		Drinking water from a cup rinsed the cup before pouring some water into it from the plastic bottle	Water	
1:25	W		Serving kunu into the cup used in drinking water	Kunun gyada	
1:25	C		Sleeping under the tree		
1:30	W		Drinking kunu from the cup	Kunun gyada	
1:31	W		Drinking kunu	Kunun gyada	
1:35	C		Still sleeping under the tree		
1:35	W		Washing the cup used for drinking kunu		
1:40	W		Farming		
1:40	C		Sleeping		
1:45	W		Farming		
1:50	W		Finishing up from the farm		
1:55	W	C	Washing hands with water		
1:55	W		Packing stuff into the large utensil she came to the farm		
2:00	W	C	Carrying baby on her back. Carry the large utensil on her head.		
2:05	W		Climbing motorcycle to go back home while carrying baby on her back and carrying utensil on her head		
2:10	W	C	Carrying baby on her back on motorcycle		
2:15	W	C	Carrying child on her back on motorcycle		
2:20	W	C	Carrying baby on her back on motorcycle		
2:25	W		Carrying utensil into the house		
2:25	W	C	Keeping the child under the shade to play		

TIME	ACTOR INITIALS	RECIPIENT INITIALS	WHAT HAPPENED	FOOD	Reactivity
2:25	C1	W	Welcoming mother from the farm		
2:30	W		Mother is taking a bath		
2;35	C		Playing under the shade		
2:35	C1	C	Playing with the baby under the shade		
2:40	W		Starting the fire to boil water	Water	
2:45	W	C	Carrying baby on her lap		
2:45	W	C	Breastfeeding baby	Breast milk	
2:50	W	C	Breastfeeding baby	Breast milk	
2:55	C		Playing on mother lap while breastfeeding	Breast milk	
3:00	W		Checking firewood		
3:05			Adding noodles to the boiling water on the fire	Spaghetti	
3:10	W		Food is ready to be served	Spaghetti	
3:10	W		Serving food into food flask	Spaghetti	
3:15	W		Adding palm oil into pot on the fire	Palm oil	
3:20	W		Adding onions into the palm oil on the fire	Palm oil onions	
3:25	W		Bringing down fried onions with palm oil from the fire	Palm oil onions	
3:30	W		Washing hands after preparing		
3:30	W		Washing utensils used in preparing the meal		
3:35	C		Playing on the mat under the shade with older daughter		
3:40	W		Serving spaghetti with palm oil and onions into a bowl.	Spaghetti palm oil onions	
3:41	W		Adding salt into spaghetti and	Spaghetti	

TIME	ACTOR INITIALS	RECIPIENT INITIALS	WHAT HAPPENED	FOOD	Reactivity
			mixing	, palm oil, onions	
3:42	W	C	Eating together with older daughter and feeding the baby	Spaghetti	
3:43	C		Eating by herself from the mother bowl		
3:44	W	C	Assisting the child to eat	Spaghetti	
3:45	W		Eating together with the older daughter from the same bowl	spaghetti	
3:49	C		Eating with her hands from the mothers	Spaghetti	
3:50	W		Washing the baby hands and mouth with water		
3:50	C1		Washing hands with soap and water		
3:50	W	C1	Supervising the older daughter washing her hands		
4:00	W	C	Giving the child water to drink from a cup	Water	
4:05	C1		Drinking water from the clay pot in front of a room with a cup	Water	
4:10	W		Sitting on a mat under the shade in front of the room		
4:15	W	C	Playing with the child on the mat		
4:20	C1	C	Older daughter playing with the child on the mat under the tree		
4:25	C		Lying down on the mat beside the mother under the shade		
4:30	C1	C	Playing with the child on the mat. Tickling the child on the stomach		
4:30	C		Playing and laughing with older daughter on the mat		
4:35	C		Playing on the mat with the mother and older child		
4:40	W	C,C1	Playing with older daughter and child on the mat under the shade		
4:45	W		Carrying the child on her laps		
4:45	W	C	Breastfeeding the child on her laps	Breast	

TIME	ACTOR INITIALS	RECIPIENT INITIALS	WHAT HAPPENED	FOOD	Reactivity
				milk	
4:50	W	C	Breastfeeding the child	Breast milk	
4:55	W	C	Breastfeeding the child	Breast milk	
5:00	W	C	Breastfeeding the child	Breast milk	
5:05	W		Starting the fire		
5:10	W		Starting the fire		
5:10	W		Warming beans pudding on the fire in a pot with water	Alele (beans pudding)	
5:15	W		Waiting for the beans pudding to warm up		
5:20	W		Serving warm allele into a food flask	Beans pudding	
5:25	W	C	Washing the child hands with soap and water		
5:25	C1		Washing her hands with water		
5:30	W	C	Assisting the baby to eat from the same bowl with the older child	Beans pudding	
5:31	C1		Eating from the same bowl with the baby	Beans pudding	
5:32	C1	C	Assisting the baby to eat. the baby accept food from her	Beans pudding	
5:35	C		Eating with her hands from the same bowl with her older sister	Beans pudding	
5:40	W	C	Bathing the child		
5:45	W	C	Bathing the child		
5:50	W	C	Dressing the child		
5:55	W	C	Carrying the child on her back		
6:00	W	C1	Chatting with the older daughter under the shade		
6:05	W	C1	Chatting with older daughter under the shade		

TIME	ACTOR INITIALS	RECIPIENT INITIALS	WHAT HAPPENED	FOOD	Reactivity

TIME	ACTOR INITIALS	RECIPIENT INITIALS	WHAT HAPPENED	FOOD	Reactivity

PARTICIPANT ID: __Mud house with zinc door _______

Primary Questions (Complete after you complete data collection):

From what you have seen observing THIS HOUSEHOLD, please answer the following questions in detail:

Question	Write your <u>detailed</u> response here
1. In this household, what would be the best way(s) to give nutritional supplements such as an MNP to young children to ensure compliance?<br><br>Why?	The best way to ensure compliance is to educate the mother because the mother is always with the child. She assists the child to eat, drink and do other things.
2. In this household, who is favored during meal preparation or feeding?<br><br>How is this favoritism expressed?	The parents and older children are favored because the meal prepared and the one warmd was solid in nature
3. How could we promote the MNP supplement for this family that would attract their attention?<br><br>Who would they listen to?	MNP can be promoted to this family through the mother.<br><br>The father can also serve as influence because he might be in charge of all decisions in the household.<br><br>The religious leader can also be an avenue to promote the MNP because the religious leaders are always encouraging their members to use such product.<br>The community leaders can also be of help to promote the MNP. The only issue is they think the community leaders can use it as means of politics.
4. In this household, what would be the best ways to	The best way to limit the sharing of MNP in this household is to enlighten the mother adequately and the husband too because if

promote the MNP but limit how much it would be shared?<br><br>Why?	the mother tries to share the husband will call her attention to the fact that MNP is not for sharing.
5. What other issues and concerns did you see with this family that are important for us to consider with regard to introduction of an MNP?<br><br>Why?	The mother spent more time in the farm. The food prepared later in the day was solid in nature.<br>The child did not have a separate bowl.<br>The hygiene in the house is okay but there is room for improvement because the mother was seen serving food without washing her hands but she encouraged the children to wash their hands
6. Based on your experience observing this child today, do you believe reactivity negatively impacted your ability to see the food-related behaviors of the child?<br><br>Please explain your answer in detail.	There was no reactivity during feeding. The mother admitted that this is the usual way the child is fed and eats by herself sometimes.
